# A standardized educational Intervention to enhance acceptance and adherence to self-intermittent catheterization: Protocol for a multicenter randomized controlled trial

**DOI:** 10.1016/j.mex.2025.103557

**Published:** 2025-08-06

**Authors:** Rosario Caruso, Laura Pelizzari, Giovanni Cardilli, Simone Bajardo, Tatiana Bianconi, Maria Luisa Giordano, Giorgio Scivoletto, Alessandro Giammò, Luisa De Palma, Lina Di Lucente, Maria A. Marquez, Lisa Morbidelli, Alexandra Pellegrino, Daniela Monducci, Antonio Boffa, Stefania Musco

**Affiliations:** a Department of Biomedical Sciences for Health, University of Milan, Milan, Italy; b Department of Rehabilitative Medicine, AUSL Piacenza, Fiorenzuola d'Arda, Piacenza, Italy; c Santa Lucia Foundation IRCCS, Rome, Italy; d IRCCS Ospedale Sacro Cuore Don Calabria, Negrar, Verona, Italy; e Spinal Cord Unit, ASST GOM Niguarda, Milan, Italy; f Service of Occupational Therapy, Orthopedic Trauma Center, Rome, Italy; g Unit of Neuro-Urology, Città della Salute e della Scienza University Hospital, University of Turin, Turin, Italy; h Spinal Cord Unit, Policlinico University Hospital, Bari, Italy; i Universidad Fernando Pessoa-Canarias, Las Palmas, Spain; j Neuro-Urology Department, Careggi University Hospital, 50134 Firenze, Italy; k Montecatone Rehabilitation Institute, Imola, Italy

**Keywords:** Intermittent catheterization, Patient education, Acceptance, Adherence, Randomized controlled trial, Neurogenic bladder, Self-catheterization, Patient-reported outcomes, Assistive technology, Rehabilitation

## Abstract

Intermittent catheterization (IC) is the gold-standard management for individuals with non-obstructed urinary retention and neurogenic bladder. Learning IC is physically and psychologically challenging, and several men express fear of practicing IC because of the risk of urethral trauma and urinary infections. This study aims to evaluate the effectiveness of an enhanced educational intervention in improving IC acceptance and adherence compared to standard clinical practice training. This multicenter, parallel-group, randomized controlled trial will enroll 128 male patients, randomly assigned to either the intervention group, receiving standard training plus an informational booklet and video tutorial, or the control group, receiving standard training alone. The primary outcome is the improvement in IC acceptance, measured by the Intermittent Catheterization Acceptance Test (I-CAT) score at three months (T2). Secondary outcomes include adherence levels (I-CAS), urinary symptoms (USQNB-IC), procedural autonomy (Autonomy Checklist), and patient-reported satisfaction (QUEST). Findings from this study will provide evidence-based guidance on optimizing IC education to enhance patient self-management and adherence.

## Highlights

• Evaluates an enhanced educational intervention to improve IC acceptance and adherence.
• Randomized controlled trial design with three follow-up time points (T0, T1, T2).
• Primary outcome: I-CAT score improvement at three months (T2).

## Graphical abstract

**Figure:**
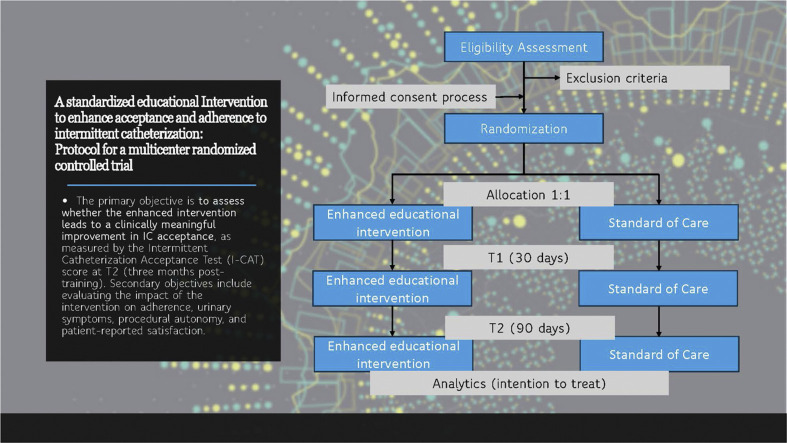

Specifications table

*This table provides general information on your protocol.*

**Table:** 

**Subject area**	Medicine and Dentistry
**More specific subject area**	*Neuro-urology; nursing; rehabilitation*
**Name of your protocol**	A standardized educational Intervention to enhance acceptance and adherence to intermittent catheterization: Protocol for a multicenter randomized controlled trial (Serendipity protocol)
**Reagents/tools**	Not applicable; intervention consists of educational materials (informational booklet, video tutorial) and standard IC training. Catheters used include no-touch sheaths and protective tip catheters (manufacturer details per clinical setting).
**Experimental design**	A multicenter, parallel-group, randomized controlled trial (RCT) comparing standard clinical training with an enhanced educational intervention. Patients are randomly assigned to Group A (enhanced training) or Group B (standard training), with follow-ups at T0 (baseline), T1 (1 month), and T2 (3 months). The primary outcome is IC acceptance (I-CAT score at T2), with secondary outcomes including adherence (I-CAS), urinary symptoms (USQNB-IC), procedural autonomy (Autonomy Checklist), and satisfaction (QUEST).
**Trial registration**	This trial was registered at ClinicalTrials.gov (identifier: NCT07084376)
**Ethics**	The Comitato Etico Regione Toscana approved this study on February 4, 2025 - Area Vasta Centro (Ethical Committee of the promoting center, Azienda Ospedaliero-Universitaria Careggi, Firenze), protocol number: 27,679_spe. Written informed consent will be obtained from all participants before enrollment. The study follows Good Clinical Practice (GCP) guidelines, the Declaration of Helsinki, and EU GDPR for data protection.
**Value of the Protocol**	• To Provide a structured, evidence-based educational intervention to improve IC acceptance and adherence among male patients. • To Standardize IC training across multiple centers using validated tools (I-CAT, I-CAS, USQNB-IC, QUEST). • To Enhance patient self-management and autonomy by integrating behavioral health strategies, assistive technology, and patient-centered training approaches.

## Background

Intermittent catheterization (IC) is a widely used and clinically effective method for managing non-obstructive rinary retention and neurogenic bladder, particularly in individuals with spinal cord injuries, multiple sclerosis, and other neurological conditions [[1], [2], [3]]avoiding upper urinary tract complications and improving urinary symptoms, and quality of life [4,5]. However, despite its benefits, adherence to IC remains a significant challenge. Many patients struggle with psychological discomfort, procedural difficulties, and inadequate training, all of which can negatively impact their acceptance and consistent use of IC [6,7].

Educational interventions play a critical role in addressing these barriers and improving adherence to IC [4]. Existing studies highlight the importance of providing structured training to help patients integrate IC into their daily routines effectively [6,8]. However, the optimal method for educating patients remains undetermined, as educational practices vary widely across healthcare settings. There is currently no standardized approach to enhancing patient acceptance and adherence, underscoring the need for evidence-based educational strategies [2,4,7,9].

This protocol describes a multicenter, parallel-group, randomized controlled trial (RCT) designed to evaluate the effectiveness of an enhanced educational intervention for patients learning IC. The intervention consists of standard clinical training supplemented with an informational booklet and video tutorial to reinforce key concepts. The study will measure changes in IC acceptance using the Intermittent Catheterization Acceptance Test (I-CAT) alongside short-term adherence, urinary symptoms, functional autonomy, and patient satisfaction [10,11]. In other words, this protocol aims to provide a replicable methodology for improving IC training in clinical practice by standardizing and systematically evaluating this educational intervention. The results from this study may help to refine patient education strategies, ultimately improving adherence and patient-reported outcomes associated with IC [3,12].

## Description of protocol

### Study design

This study is a multicenter, parallel-group RCT conducted across 17 outpatient and hospital settings. The trial aims to evaluate the effectiveness of an enhanced educational intervention on IC acceptance compared to standard clinical practice training. Participants will be adult male patients (>18 years old) requiring IC training who meet the inclusion criteria and provide informed consent. This trial was registered at ClinicalTrials.gov (identifier: NCT07084376) before participant enrollment.

Participants will be randomly assigned (1:1) to the intervention group (Group A) or the control group (Group B) using a computer-generated list. Group A will receive standard clinical training plus an informational booklet and video tutorial, while Group B will receive standard training only.

The study follow-up period will be three months, with assessments conducted at three time points: baseline (T0), one-month post-initial training (T1), and three months post-initial training (T2). The primary outcome is the change in I-CAT scores, assessing patient acceptance of IC. Secondary outcomes include adherence to IC, urinary symptoms, procedural autonomy, patient-reported satisfaction, and functional abilities.

The study is open-label to both participants and investigators, as self-reported outcome measures will be used. However, the statistician analyzing the data will remain blinded to group assignments. Ethical approval has been obtained from institutional review boards, and all participants will provide written informed consent before enrollment. Data will be securely stored following GDPR regulations, with de-identified information retained for 15 years.

### Settings

The study will be conducted in both outpatient and hospitalized settings across multiple centers, ensuring a diverse patient population and enhancing the generalizability of the findings. The coordinating center for this study is the Azienda Ospedaliero-Universitaria di Careggi in Firenze, which will oversee the study’s implementation, adherence to the protocol, and data collection processes across all participating sites.

This list includes centers that have expressed their commitment to the study, but it is not exhaustive. Additional centers may join as the study progresses, and any updates regarding participating institutions will be incorporated into the protocol accordingly.

### Participant recruitment and eligibility

Patients referred for IC training will undergo an initial screening to determine their eligibility based on predefined inclusion and exclusion criteria. Those who meet the eligibility requirements will be invited to participate and provided with comprehensive information about the study. Written informed consent will be obtained from all eligible participants before enrollment. Once consent is obtained, baseline data collection will be conducted, including sociodemographic information, clinical history, and prior experience with IC.

The study will include naïve patients who require training in IC using aseptic techniques, specifically focusing on using no-touch sheaths and protective tip catheters. Naïve patients are defined as those with no prior experience or training in aseptic IC or those with minimal experience (less than one month) who require structured education to perform the procedure safely. No-touch sheaths and protective tip catheters are designed to minimize contamination risks and enhance safety during catheterization. Eligible participants must be fully informed of all available catheterization devices and must voluntarily choose to use no-touch sheaths and protective tip catheters. The study will focus on adult male patients aged 18 years and older who must be capable of providing informed consent. Additionally, all participants must demonstrate a willingness to utilize no-touch sheaths and protective tip catheters as part of their educational intervention.

Exclusion criteria include cognitive impairments that may hinder comprehension of the training process, a history of anxiety, depression, or other psychiatric disorders that could impact adherence, and language barriers preventing understanding of written and verbal Italian instructions. Patients with learning difficulties affecting their ability to follow medical instructions, those diagnosed with urinary tract neoplasms or penile/urethral anomalies, and those requiring two or fewer catheterizations per day—making them unsuitable for adherence evaluation—will also be excluded from the study.

### Sampling strategy

The study will utilize a consecutive sampling approach to recruit participants. This method involves selecting every eligible patient who meets the inclusion criteria and consents to participate until the required sample size is achieved. Once enrolled, participants will be randomly assigned to either Group A (intervention group) or Group B (control group) using a computer-generated randomization sequence.

This approach ensures that the sample accurately represents the diversity of the patient population typically seen in clinical settings while maintaining the practicality and efficiency needed in busy healthcare environments. The consecutive sampling aims to minimize the risk of selection bias, which may arise from convenience-based recruitment, while ensuring that enrollment strictly follows the eligibility criteria.

### Sampling procedure

The screening process will involve evaluating patients based on the predefined inclusion and exclusion criteria. This will include a review of medical records and an initial assessment to determine each patient’s eligibility for participation in the study. Once deemed eligible, patients will be approached consecutively and invited to participate. The recruitment process will continue until the required sample size is reached, ensuring that the study population is representative of the broader clinical population and diverse in terms of patient characteristics.

Before enrollment, written informed consent will be obtained from all participants. Patients will receive detailed information about the study, including its purpose, procedures, potential risks, and expected benefits. They will have the opportunity to ask questions before deciding whether to participate. Participation is entirely voluntary, and patients may withdraw from the study at any time without any impact on their standard medical care. After enrollment, participants will be assigned to one of the two study groups.

While contamination between groups is theoretically possible, it would likely lead to an underestimation of the intervention effect rather than an artificial inflation, as the intervention group receives all components provided to the control group plus additional materials. To mitigate this risk, participants were clearly instructed not to share training details or educational materials with others. Moreover, supplementary materials were distributed individually and not made publicly accessible, and educational sessions were scheduled to minimize overlap and patient interaction.

### Randomization and allocation concealment

A randomization list of 20 cases per center, allocated in a 1:1 ratio between the intervention (Group A) and control (Group B) arms, will be created using a simple randomization algorithm. Simple randomization was deemed appropriate for this study, given the limited number of patients, ranging from 4 to 20, expected to be enrolled at each center. Unlike block randomization, which could create challenges if a center fails to complete an entire block, simple randomization ensures flexibility while maintaining methodological rigor.

The randomization list will be generated independently by the methodologist responsible for the process, ensuring no bias or influence from investigators. This list will be securely stored and concealed from the principal investigator (PI) and study centers to maintain strict allocation concealment.

The randomization process will employ a sealed, opaque envelope system to ensure allocation concealment. For each center, 20 envelopes will be prepared, corresponding to the 20 cases on the randomization list. Each envelope will be sequentially numbered from 1 to 20 and will contain the group assignment (Group A or Group B) based on the randomization list. The envelopes will be opaque and sealed to prevent any possibility of visual identification of group assignments before opening.

Once prepared, the envelopes will be sent to each participating center in a tamper-proof package by the methodologist who created the randomization list. The randomization list will remain securely stored, ensuring that neither the PI nor the study sites have access to it. At each center, envelopes will be opened sequentially and in numerical order by a designated study coordinator after confirming the patient’s eligibility and obtaining written informed consent. The study coordinator will be trained to ensure compliance with the protocol and will have no involvement in delivering the intervention or assessing outcomes, maintaining a clear separation of roles and minimizing bias.

All randomization and allocation processes will be systematically documented. Once an envelope is opened, the group assignment will be recorded, and the envelope will be securely stored to maintain an audit trail. The randomization and envelope-opening processes will be periodically audited to ensure adherence to the protocol and to detect any deviations that may compromise allocation concealment.

### Sample size

The sample size for this study was determined to ensure sufficient statistical power to detect a meaningful difference in median I-CAT scores between the intervention and control groups. The primary endpoint aims to demonstrate an improvement in median I-CAT scores from 3.0 to 3.5, which corresponds to an increase from the median acceptance score to the 75th percentile of the previously reported distribution [10,11].

To achieve this, a significance level of 0.05 (5 %) was selected, meaning there is a 5 % probability of a Type I error, where a difference is incorrectly concluded when none exists. A power of 80 % (0.80) was chosen, ensuring that the study has an 80 % probability of detecting a true difference between the groups if one exists, thereby reducing the risk of a Type II error. Based on previous studies and pilot data, a standard deviation of 1 was assumed for the calculation [10,11]. Given these parameters, the sample size calculation was performed using a non-parametric (Mann-Whitney U) test to compare the two independent groups.

The formula used for calculating the required sample size accounts for effect size, significance level, and power. The required sample size per group, denoted as n, is calculated using the equation:

(1) $$n=\frac{{\left(Z_{\alpha /2}+Z_{\beta }\right)}^{2}\times \;2\;\times \sigma^{2}}{\Delta^{2}}$$

In this approach, Z_α/2_ represents the Z-score for the chosen significance level (1.96 for α = 0.05), Z_β_ is the Z-score for the desired power (0.84 for 80 % power), σ² represents variance, and Δ is the effect size, defined as the difference between the 75th percentile and the median of the I-CAT score distribution. The calculations indicate that 64 participants per group are required to achieve the desired statistical power and significance level, resulting in a total sample size of 128 participants. This sample size is based on an effect size of 0.5, calculated as the difference between the two medians divided by the standard deviation. A sensitivity analysis was performed to evaluate how variations in the expected effect size impact the required sample size. For smaller effect sizes ranging from 0.5 to 0.2, the required sample size per group increased, ranging from 64 to 392 participants per group. Given the study objectives and feasibility considerations, the target sample size was determined, assuming an effect size of 0.5, confirming that 64 patients per group are sufficient to ensure reliable and statistically meaningful results.

### Primary endpoint

The primary endpoint of this study is the improvement in median I-CAT scores at T2 (three months post-initial training), assessing IC acceptance. A 0.5-point increase in the median I-CAT score (from 3.0 to 3.5) represents a shift from the median to the 75th percentile of the previously reported distribution [10,11]. This reduction in IC-related reluctance is considered clinically meaningful, indicating a higher level of acceptance among participants receiving the enhanced educational intervention compared to those undergoing standard clinical practice training. The study is powered to detect this difference at T2, with 80 % power and a 5 % significance level, ensuring that the observed effect is both statistically and clinically significant.

### Procedures

The study will begin with a screening process using a consecutive sampling approach to ensure that all eligible individuals are considered for participation. Patients will be evaluated based on the inclusion and exclusion criteria, and those who meet the eligibility requirements will be invited to participate. Prior to enrollment, patients will receive comprehensive information about the study’s purpose, procedures, potential risks, and benefits. After having the opportunity to ask questions, those willing to participate will provide written informed consent before proceeding further.

To maintain methodological rigor, randomization will be conducted using a block randomization method with allocation concealment via sealed opaque envelopes, ensuring an unbiased assignment of participants to either study arm.

All participants must sign a written informed consent form before engaging in the study. They will be assured that their participation is voluntary and that they have the right to withdraw at any time without any consequences for their standard medical care. Further details regarding follow-up assessments are described in the section on Follow-ups.

### Enhanced educational intervention (Group A)

Patients assigned to Group A will receive an enhanced educational intervention designed to improve their understanding and acceptance of IC. This intervention consists of standard clinical practice training supplemented with an informational booklet and a video tutorial. The educational materials will provide detailed instructions on the proper use and benefits of IC. The primary goal of this enhanced intervention is to empower patients by providing them with knowledge and skills that enable them to perform intermittent catheterization effectively and confidently, ultimately improving adherence and acceptance rates.

### Standard clinical practice training (Group B)

Patients in Group B will receive standard clinical practice training, which consists of basic instructions and demonstrations provided by healthcare professionals on how to perform intermittent catheterization correctly. This training will focus primarily on the technical aspects of catheterization, including proper aseptic techniques, insertion and removal procedures, and hygiene practices to prevent infections. However, this training will not include the additional educational materials provided to Group A, such as the informational booklet and video tutorial on the no-touch sheath and protective tip catheters. While the standard training ensures that patients receive essential knowledge on catheterization, it does not provide reinforced learning tools designed to enhance acceptance and adherence, as in the enhanced intervention.

### Follow-ups

Data collection will occur at three key time points throughout the study. The initial training session (T0) will serve as the baseline assessment. The first follow-up (T1) will be conducted one month after the initial training to assess early outcomes, while the second follow-up (T2) will take place three months after the initial training to evaluate short-term effects. These follow-up assessments will allow for the monitoring of changes in acceptance, adherence, and patient-reported outcomes over time.

### Measurements

Sociodemographic and clinical data will be collected to comprehensively understand the patient population and the factors influencing adherence and acceptance of IC.

Sociodemographic data will include age, gender, educational level, employment status, marital status, and socioeconomic status. Age will be recorded to analyze the distribution of the patient population and its potential impact on IC adherence. Although the study focuses on male patients, gender-related data may still be relevant for broader analyses [13]. The educational level will be documented as it may influence the patient’s understanding of medical instructions and adherence to IC routines. Employment status will be considered, as work-related stress and availability could impact adherence, while marital status will be evaluated to assess the role of social support in adherence and overall well-being. Socioeconomic status will also be examined, as economic factors may influence access to healthcare resources and compliance with prescribed treatments.

Clinical data will include primary diagnosis, condition duration, previous treatments, comorbidities, medication use, urinary function, and prior IC experience. The type of Neurological disease in patients having neurogenic bladder will be recorded, identifying whether it results from conditions such as spinal cord injury, multiple sclerosis or others. The duration of the condition will be noted, as the length of time since diagnosis may impact the patient’s adaptation to IC. A detailed history of previous treatments will be collected, including surgeries, medications, and prior catheterization experiences. Comorbidities such as diabetes or cardiovascular diseases will be assessed to determine their potential impact on overall health and IC adherence. Medication use, particularly drugs related to urinary symptoms, will be recorded to understand their effect on catheterization efficacy and adherence. Baseline urinary function will be evaluated to correlate with changes observed throughout the study. Additionally, previous IC experience, if any, will be documented to provide context for current acceptance and adherence levels.

Self-report assessments will be administered to evaluate different aspects of the patient’s experience, adherence, and outcomes related to IC. All measurement tools selected for this study are supported by evidence of validity and reliability:

(a) The Intermittent Catheterization Acceptance Test (I-CAT) will be administered at baseline (T0), one month post-initial training (T1), and three months post-initial training (T2). The I-CAT is designed to measure changes in acceptance rates of IC over time. It evaluates psychological comfort, willingness, and overall acceptance, helping to assess how well patients adapt to the procedure and the impact of educational interventions on their acceptance levels [10].
(b) The Urinary Symptoms Questionnaire for Neurogenic Bladder in IC (USQNB-IC) will be conducted at T0, T1, and T2. This instrument evaluates urinary symptoms in patients with neurogenic bladder undergoing IC. It measures the severity and frequency of symptoms, including incontinence, urgency, and discomfort, allowing for the monitoring of symptomatic changes and potential improvements in urinary health with consistent IC use [14].
(c) The Intermittent Catheter Adherence Scale (I-CAS) will be administered at T1 and T2 to measure adherence to the prescribed IC regimen. This tool assesses the frequency and consistency of catheter use as recommended by healthcare providers. Evaluating adherence through I-CAS will help identify barriers to consistent IC and assess the effectiveness of educational interventions in improving compliance [11].
(d) The Autonomy Checklist will be completed at T0, T1, and T2 to assess procedural autonomy in IC. This checklist evaluates the patient’s ability to independently manage catheterization, including preparation, insertion, and hygiene maintenance. High scores indicate successful training and an increase in self-sufficiency in performing IC.
(e) The Serendipity Questionnaire, developed by the researchers involved in this research, will be administered at T2 to capture subjective experiences and unexpected positive outcomes during the IC journey. This questionnaire includes items that explore patient insights, satisfaction, and any unexpected benefits they may have encountered. The qualitative data obtained will provide a richer understanding of the patient experience beyond clinical measures.
(f) The Quebec User Evaluation of Satisfaction with Assistive Technology (QUEST) will be administered at T1 and T2 to assess patient satisfaction with the assistive devices used for IC. This instrument includes questions about the ease of use, effectiveness, and overall satisfaction with the catheter and related equipment. By identifying areas for improvement in device design and patient training, QUEST ensures that tools meet the practical needs of patients [15].
(g) Motor function assessments will also be included in the study. The Motor Assessment using a Dynamometer will be conducted at T0 and T1 to measure grip strength, an essential factor in the ability to perform IC independently [16]. This test will help determine whether physical limitations may impact a patient’s ability to handle and use the catheter effectively by evaluating motor strength. The Manual Dexterity Assessment using the Nine Hole Test will be performed at T0 and T1 to measure fine motor skills, which are crucial for successful IC execution [17]. This test requires participants to place and remove pegs from a board, providing a quantitative measure of hand function. It will help identify patients who may require additional support or adaptive techniques to manage IC effectively.

### Statistical plan

The statistical analysis of this study will begin with descriptive statistics to summarize the baseline characteristics of participants, including sociodemographic and clinical data, under intention-to-treat analysis. Continuous variables will be presented using means, medians, standard deviations, and interquartile ranges, depending on their distribution. Categorical variables will be summarized using frequencies and percentages.

Inferential statistical analyses will be conducted to compare outcomes between the enhanced educational intervention group and the standard clinical practice training group. Independent *t*-tests will be used to compare normally distributed continuous variables, while Mann-Whitney U tests will be applied for skewed distributions and for the primary outcome (I-CAT score at T2). Chi-square tests will be used to compare categorical variables between the study groups.

Correlation analyses will be conducted following these comparisons to explore relationships between key study variables. Specifically, associations between IC acceptance scores (I-CAT), adherence levels (I-CAS), urinary symptoms (USQNB-IC), and functional abilities (Autonomy Checklist and motor assessments) will be examined. Depending on the distribution of the data, either Pearson’s correlation coefficient (for normally distributed variables) or Spearman’s rank correlation coefficient (for non-normally distributed variables) will be used. These analyses will help identify how different aspects of the patient experience and clinical characteristics interrelate, providing insight into the factors influencing IC acceptance and adherence.

To further investigate factors associated with adherence and secondary outcomes, multivariable analyses will be performed. Logistic regression models will be used to analyze binary outcomes, such as adherence status, while linear regression models will be applied to continuous outcomes, including I-CAT scores. Predictor variables will include sociodemographic factors (e.g., age, education level, socioeconomic status), clinical characteristics (e.g., primary diagnosis, comorbidities), and intervention type (enhanced educational intervention vs. standard clinical training). Interaction terms will be incorporated to assess whether the effects of the intervention differ across subgroups, ensuring a comprehensive evaluation of potential effect modifiers. Model selection will be guided by theoretical considerations and statistical criteria, using stepwise or backward elimination methods to refine the models.

Handling missing data will be an important aspect of the analysis. This study assumes that data are missing at random (MAR), meaning that missingness is related to observed data but not to unobserved data [18]. All instances of missing data will be identified and documented, and patterns of missingness will be analyzed to determine whether they are systematically related to specific variables. Descriptive statistics will be used to summarize the extent and distribution of missing data for each variable.

Multiple imputation will be used as the primary method for handling missing data to minimize bias and preserve statistical power [19]. This approach generates multiple plausible datasets by imputing missing values based on observed data, which are then analyzed separately. The results from these datasets will be combined to produce estimates and confidence intervals that appropriately reflect the uncertainty introduced by missing data. Multiple imputations using chained equations will be performed, suitable for handling various variables, including continuous, binary, and categorical data.

To evaluate the robustness of the findings, sensitivity analyses will be conducted, comparing the results of multiple imputations with alternative approaches, such as complete case analysis (where only participants with complete data are analyzed) and single imputation methods [18]. These analyses will ensure that the handling of missing data does not introduce bias into the study conclusions. The extent of missing data, the imputation methods applied, and the results of sensitivity analyses will be transparently reported in all study publications and presentations to ensure scientific integrity and reproducibility.

All statistical analyses will be performed using R (R Core Team, 2023). The significance level (alpha) for hypothesis testing will be set at 5 % (0.05). When multiple comparisons are conducted, appropriate adjustment procedures will be applied to control for Type I error inflation. Two-tailed tests will be employed for all analyses.

## Ethical considerations

This study has been approved by the Ethical Committee of the promoting center, Comitato Etico Regione Toscana - Area Vasta Centro, which reviewed and issued a favorable opinion on February 4, 2025 (protocol number: 27,679_spe). The Azienda Ospedaliero-Universitaria Careggi in Firenze is the promoting center and ensures compliance with ethical and regulatory guidelines throughout the study.

The research will be conducted in accordance with the Declaration of Helsinki, Good Clinical Practice (GCP) guidelines, and national regulations governing clinical studies. Participation will be entirely voluntary, and patients will have the right to withdraw at any time without any consequences for their standard medical care.

The study will also comply with European Union General Data Protection Regulation (EU GDPR) standards to safeguard participant privacy and data confidentiality. De-identified data will be securely stored, and access will be restricted to authorized personnel. The Ethical Committee reserves the right to monitor the study throughout its duration to ensure compliance with ethical principles and best research practices.

## Protocol validation

Although no preliminary results are currently available, the hypothesis of a 0.5-point improvement in the I-CAT score at T2 is grounded in prior research and clinical reasoning. The assumption that the enhanced educational intervention will lead to a clinically meaningful increase in IC acceptance is supported by existing evidence on patient education and adherence to self-catheterization.

The I-CAT has been validated as a reliable measure of patient acceptance, with previous studies indicating that structured training and educational interventions significantly improve adherence and psychological comfort with IC [10]. The expected increase from a median I-CAT score of 3.0 to 3.5 represents a shift toward higher acceptance levels, aligning with improvements observed in studies evaluating similar patient-centered educational programs [20].

Moreover, previous research on adherence to self-catheterization suggests that educational reinforcement and patient-centered training approaches can lead to measurable improvements in acceptance and compliance [[21], [22], [23]]. The study design, which integrates a video tutorial and an informational booklet alongside standard training, is based on established methods that have been shown to enhance patient confidence, self-efficacy, and long-term adherence [24].

Additionally, the sensitivity analysis conducted during sample size estimation supports the feasibility of detecting this hypothesized effect. If the enhanced intervention successfully reduces negative perceptions and barriers associated with IC, a 0.5-point improvement in the median I-CAT score should be observable by T2.

The plausibility of this effect size is further reinforced by the structured follow-up assessments at T1 and T2, ensuring that the intervention’s impact on acceptance is tracked over time. Once data collection begins, adherence rates, patient-reported experiences, and I-CAT score distributions will provide further insights into the validity of the assumed effect size.

## Limitations

Several limitations should be acknowledged regarding this protocol, which resulted in the tradeoff between feasibility and optimal solutions to mitigate the risk of biases. One potential limitation is the reliance on self-reported measures, such as the I-CAT and the I-CAS. Self-reported data may be subject to social desirability bias or recall bias, potentially affecting the accuracy of adherence and acceptance measurements. While validated tools are used to mitigate these issues, objective adherence measures, such as tracking actual catheter use, are not included in the protocol. Another limitation is the follow-up period of three months (T2), which may not fully capture long-term adherence and sustained acceptance of intermittent catheterization (IC). Although three months provides insight into short- and medium-term outcomes, future studies with extended follow-up periods may be needed to assess long-term patient adherence and potential changes in acceptance beyond the study period. The study also focuses exclusively on male patients, which limits the generalizability of findings to female populations. While this design choice was made to reduce variability in anatomical and procedural differences, future research should evaluate whether the educational intervention is equally effective in female patients requiring IC.

Additionally, the multicenter design introduces variability in clinical practice settings, patient populations, and training approaches, which may impact intervention fidelity. Although efforts will be made to standardize training and ensure protocol adherence across study sites, minor differences in educational delivery and patient interactions could influence outcomes. Finally, while the sample size calculation is based on a clinically meaningful effect size (0.5-point increase in I-CAT scores at T2), the study may be underpowered to detect more minor but still relevant improvements. Sensitivity analyses have considered a range of effect sizes, but if the actual effect is smaller than anticipated, the study may lack sufficient power to demonstrate statistical significance. Although the sample size is adequately powered to detect the primary outcome, caution should be exercised in generalizing the results to broader populations, especially given the inclusion of only male patients and the potential variability across clinical centers.

Despite these limitations, the study design, including randomization, allocation concealment, and the use of validated measurement tools, strengthens the internal validity of the findings and provides a robust framework for evaluating the impact of an enhanced educational intervention on IC adherence and acceptance.

## CRediT authorship contribution statement

**Rosario Caruso:** Conceptualization, Writing – original draft. **Laura Pelizzari:** Conceptualization, Validation, Writing – review & editing. **Giovanni Cardilli:** Conceptualization, Validation, Writing – review & editing. **Simone Bajardo:** Conceptualization, Validation, Writing – review & editing. **Tatiana Bianconi:** Conceptualization, Validation, Writing – review & editing. **Maria Luisa Giordano:** Conceptualization, Validation, Writing – review & editing. **Giorgio Scivoletto:** Conceptualization, Validation, Writing – review & editing. **Alessandro Giammò:** Conceptualization, Validation, Writing – review & editing. **Luisa De Palma:** Conceptualization, Validation, Writing – review & editing. **Lina Di Lucente:** Conceptualization, Validation, Writing – review & editing. **Maria A. Marquez:** Conceptualization, Validation, Writing – review & editing. **Lisa Morbidelli:** Conceptualization, Validation, Writing – review & editing. **Alexandra Pellegrino:** Conceptualization, Validation, Writing – review & editing. **Daniela Monducci:** Conceptualization, Validation, Writing – review & editing. **Antonio Boffa:** Conceptualization, Validation, Writing – review & editing. **Stefania Musco:** Conceptualization, Writing – original draft.

## Declaration of competing interest

The authors declare that they have no known competing financial interests or personal relationships that could have appeared to influence the work reported in this paper.

## Data Availability

No data was used for the research described in the article.
